# The Uncharted Link Between Desogestrel and Hypercalcemia

**DOI:** 10.7759/cureus.40577

**Published:** 2023-06-17

**Authors:** Nishka Utpat, Radhika Annam, Smita Kargutkar-Ajgaonkar

**Affiliations:** 1 Research, Rutgers Health/Community Medical Center, Toms River, USA; 2 Endocrinology, Diabetes, and Metabolism, Hospital Sisters Health System Medical Group, Effingham, USA; 3 Endocrinology, Diabetes, and Metabolism, Ace Endocrinology, Red Bank, USA

**Keywords:** vitamin d, parathyroid hormone, calcium levels, calcium metabolism, oral contraceptives, desogestrel, hypercalcemia

## Abstract

This report presents a unique case of hypercalcemia with an elusive etiology. A 37-year-old Caucasian female with a history of gonadotropin-secreting pituitary microadenoma and recurrent nephrolithiasis was found to have hypercalcemia, hypercalciuria, elevated 1,25-dihydroxyvitamin D levels, and low parathyroid hormone levels. Extensive investigations were conducted to identify the cause, including ruling out sarcoidosis and other granulomatous disorders. Imaging and diagnostic testing revealed normal results. The patient’s condition considerably improved after the cessation of an oral contraceptive pill containing desogestrel. This surprising association raises the possibility that the use of desogestrel could result in hypercalcemia as one of the side effects. To ensure proper care and avoid consequences linked to severe hypercalcemia, a high index of suspicion is needed to detect the underlying cause of hypercalcemia, even in the absence of usual indications.

## Introduction

Hypercalcemia is a medical condition characterized by high levels of calcium in the blood (normal range: 2.1-2.6 mmol per liter). It is seen in 1% of the general population and about 2% of cancer patients, and it can lead to various complications, such as cardiac arrhythmia, neurologic complications presenting as cognitive changes, anxiety and depression, lethargy, and coma. Additionally, osteopenia, constipation, and nephrolithiasis may occur [1]. This condition commonly occurs as a result of primary hyperparathyroidism or malignancy, which accounts for over 90% of cases [2]. Other causes include familial hypocalciuric hypercalcemia and tertiary hyperparathyroidism. Also, hypercalcemia can be non-parathyroid-mediated, resulting from factors such as hypercalcemia of malignancy, vitamin D intoxication, chronic granulomatous disorders, and certain medications [3].

Granulomatous disorders, particularly sarcoidosis and tuberculosis, can also be associated with hypercalcemia [1]. In these disorders, macrophages with α-hydroxylase enzymes form granulomas, leading to the conversion of 25-hydroxyvitamin D to 1,25-dihydroxyvitamin D [4]. Elevated levels of 1,25-dihydroxyvitamin D suppress parathyroid hormone and cause hypercalcemia.

## Case presentation

The patient was a 37-year-old Caucasian female with a medical history of a gonadotropin-secreting pituitary microadenoma treated with leuprolide in the past. She also had recurrent nephrolithiasis, treated with lithotripsy in the past. Laboratory tests at the initial presentation revealed hypercalcemia, hypercalciuria, hypoparathyroidism, low 25-hydroxyvitamin D levels, elevated levels of 1,25-dihydroxyvitamin D2, normal parathyroid hormone-related peptide levels (PTH-RP), elevated serum angiotensin-converting enzyme (ACE) levels, elevated serum globulin levels, and slightly abnormal serum electrophoresis with elevated IgG and IgM (Table 1). The patient was evaluated by a hematologist to rule out monoclonal gammopathy of undetermined significance, but it was deemed unlikely to be the cause of hypercalcemia. The absence of lymphadenopathy and other signs made sarcoidosis unlikely as well. CT scans of the neck, chest, and abdomen with and without contrast did not reveal evidence of pulmonary or extrapulmonary sarcoidosis or other granulomatous disorders, except bilateral non-obstructing calculi.

**Table 1:** Patient’s laboratory test results N/A: not available; ACE: angiotensin-convertase enzyme; PTH-RP: parathyroid hormone-related peptide; iPTH: intact parathyroid hormone

Laboratory test	Patient’s values on desogestrel	Patient’s values off desogestrel	Reference range
Serum calcium, mg/dL	11	9.7	8.6-10.4
24-hour urine calcium excretion, mg	774.8	N/A	20-275
Serum iPTH, pg/mL	<6.3	12	13.8-85
25-hydroxyvitamin D, ng/mL	20.7	24.5	32-100
1,25-dihydroxyvitamin D, pg/mL	117.0	80	15-60
Serum ACE level, U/L	119	N/A	9-67
PTH-RP, pmol/L	<0.74	N/A	<2.0

**Figure 1:**
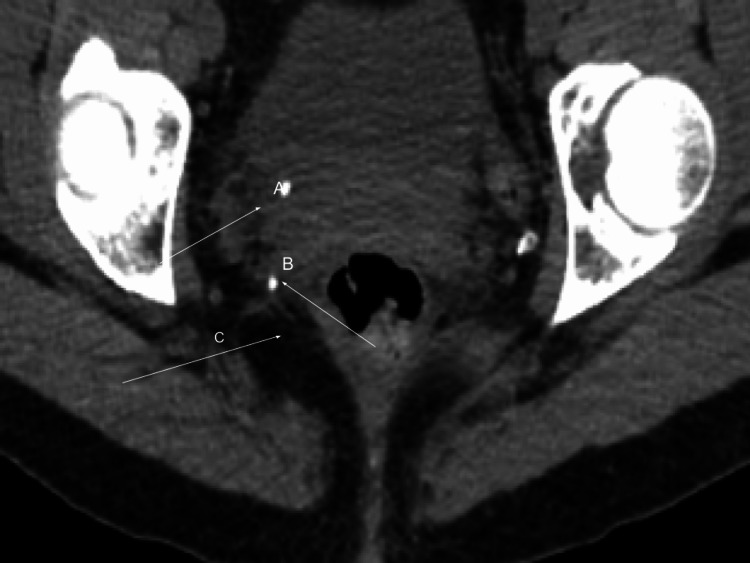
CT abdomen and pelvis without contrast The image shows 4-mm calcification in the region of the right ureterovesical Junction with right hydroureteronephrosis suspicious for obstructing renal calculus A, B: renal calculi; C: hydroureteronephrosis CT: computed tomography

**Figure 2:**
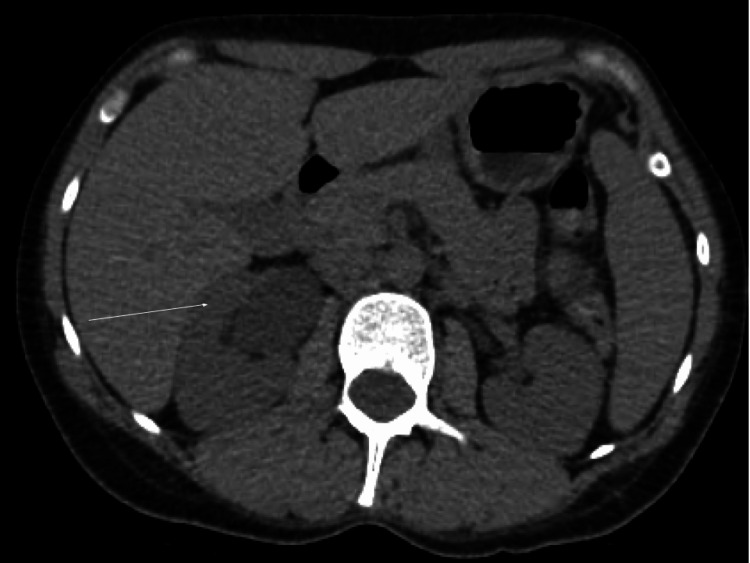
Right-sided hydroureteronephrosis raising concerns of obstructing renal calculus in the ureterovesical junction (arrow)

Figures 1-2 show the CT findings. 

A Kveim test was performed to rule out the possibility of sarcoidosis and the results were negative. The test is positive if noncaseating granulomas are found four to six weeks later. Otherwise, it is interpreted as negative. The patient was advised to undergo a positron emission tomography (PET)-CT scan to investigate the possibility of extrapulmonary sarcoidosis. However, the patient declined to undergo the scan. The only medication the patient was taking at the time of presentation was an oral contraceptive pill containing desogestrel. Her oral contraceptive was changed to one that did not contain desogestrel for a duration of three months. Following this change, her serum and urinary calcium levels, as well as her 25-hydroxyvitamin D levels, normalized. Over the course of four years, her calcium, intact parathyroid hormone (iPTH), and 1,25-dihydroxyvitamin D levels remained within the normal range. Additionally, she did not experience any recurrent nephrolithiasis.

## Discussion

The most typical presentation of hypercalcemia is an incidental finding of elevated blood calcium concentration on the biochemical screen. When symptoms do exist, they are often described as “moans, bones, stones, and groans”. Polyuria and polydipsia due to nephrogenic diabetes insipidus and intellectual obtundation may also be present in symptomatic patients. As hypercalcemia becomes more severe symptoms may manifest, such as nausea, vomiting, and QT interval shortening that might result in ventricular fibrillation, cardiac arrest, disorientation, and coma.

The two most frequent causes of hypercalcemia are primary hyperparathyroidism and secondary hyperparathyroidism, due to malignancy [3]. Tertiary hyperparathyroidism, granulomatous diseases (mostly sarcoidosis), thyrotoxicosis, medications, most notably thiazides and lithium therapy, and poorly managed therapy with alfacalcidol and calcitriol are also key causes of hypercalcemia [1]. Primary hyperparathyroidism can manifest in either a syndromic form or a nonsyndromic form such as sporadic illnesses or familial disorders. However, it can be challenging to distinguish between familial and sporadic primary hyperparathyroidism in patients as they may not have been investigated for the disease. Primary hyperparathyroidism may be caused by a de novo germline mutation in patients [5]. In granulomatous disorders, hypercalcemia arises due to the extra-renal conversion of 25-hydroxyvitamin D to 1,25-dihydroxyvitamin D by macrophage 1-α-hydroxylase within the granulomas, independent of the parathyroid hormone [2].

Patients with acute hypercalcemia frequently experience substantial volume depletion due to nausea, vomiting, and polyuria brought on by the kidney’s adverse reactions to excess calcium (reduced urine concentrating capacity and nephrogenic diabetes insipidus). Intravenous fluid administration is the initial step in the therapy to recover extracellular volume [3]. A common protocol involves giving a 1-2-liter bolus of 0.9% saline solution followed by 200-250 milliliters per hour of the same while checking calcium levels often and closely monitoring for volume overload. High-potency bisphosphonates are commonly recommended as the initial treatment of choice for hypercalcemia associated with malignancy. Osteoclasts absorb bisphosphonates, which obstruct structural proteins and cause apoptosis in these cells. Calcium levels are therefore lowered as a result of decreased bone reabsorption [6]. Calcitonin can also be used as adjunctive therapy in the early stages of treatment, along with bisphosphonates and intravenous fluids, to achieve a transient reduction in serum calcium levels [4]. Corticosteroids are sometimes used to treat malignancy-related hypercalcemia, particularly in cases of multiple myeloma, lymphomas, and breast cancer. Osteoclast activity is decreased by corticosteroids because they cause the bones to produce fewer inflammatory mediators. They can also be used to treat hypercalcemia brought on by vitamin D since they lower 1-hydroxylase activity [6]. Additionally, denosumab, an anti-resorptive agent approved for osteoporosis treatment, is recognized as a valuable adjunct in the emergency management of hypercalcemia [4].

## Conclusions

Hypercalcemia is a rare condition characterized by elevated calcium levels in the bloodstream. In the absence of evidence of sarcoidosis, it is uncommon to encounter hypercalcemia with elevated 1,25-dihydroxyvitamin D levels and low parathyroid hormone. This case touches on the possibility of extrapulmonary sarcoidosis or desogestrel-induced hypercalcemia. Physicians must maintain a high index of suspicion to identify the underlying etiology of hypercalcemia, which could enable appropriate treatment and early workup. It is important to note that the history of the patient should include the use of over-the-counter medications or herbal supplements, which might be the cause of laboratory abnormalities in a few people. Severe hypercalcemia is an endocrine emergency necessitating prompt intervention to prevent severe neurologic, cardiac, and renal complications. Finally, while there have been no cases reported of oral contraceptives containing desogestrel causing hypercalcemia, our patient’s laboratory studies normalized after stopping desogestrel, suggesting a possible correlation. However, it is important to note that the conclusions were based on a single case and should be interpreted with caution. Hence, we recommend further research to explore the relationship between desogestrel and hypercalcemia.

## References

[1] Crowley R, Gittoes N (2023). How to approach hypercalcaemia. Clin Med (Lond).

[2] Turner JJ (2017). Hypercalcaemia - presentation and management. Clin Med (Lond).

[3] Renaghan AD, Rosner M (2018). Hypercalcemia: etiology and management. Nephrol Dial Transplant.

[4] Negri AL, Rosa Diez G, Del Valle E (2014). Hypercalcemia secondary to granulomatous disease caused by the injection of methacrylate: a case series. Clin Cases Miner Bone Metab.

[5] Eastell R, Brandi ML, Costa AG, D'Amour P, Shoback DM, Thakker RV (2023). Diagnosis of asymptomatic primary hyperparathyroidism: proceedings of the Fourth International Workshop. J Clin Endocrinol Metab.

[6] Tonon CR, Silva TA, Pereira FW (2022). A review of current clinical concepts in the pathophysiology, etiology, diagnosis, and management of hypercalcemia. Med Sci Monit.

